# Increase of astrocyte apposition on GnRH neurons in early puberty onset induced by high fat diet

**DOI:** 10.1111/jne.70029

**Published:** 2025-04-15

**Authors:** Isabelle Rodrigues‐Santos, Raoni Conceição Dos‐Santos, Aline de Jesus, Rafael Appel Flores, Roberta Ribeiro Costa Rosales, Izabela Facco Caliman, Janete A. Anselmo‐Franci, José Antunes‐Rodrigues, Lucila Leico K. Elias

**Affiliations:** ^1^ Department of Physiology Ribeirão Preto Medical School, University of São Paulo Ribeirão Preto São Paulo Brazil; ^2^ Department of Cellular and Molecular Biology and Pathogenic Bioagents Ribeirão Preto Medical School, University of São Paulo Ribeirão Preto São Paulo Brazil; ^3^ Department of Basic and Oral Biology of Dentistry School of Ribeirão Preto, Laboratory of Neuroendocrinology University of São Paulo Ribeirão Preto São Paulo Brazil

**Keywords:** astrocytes, early puberty, GnRH, high‐fat diet, obesity

## Abstract

Puberty onset is driven by the activation of GnRH‐secreting neurons and can be advanced by obesity. Astrocytes are dynamic cells that react to changes in the central nervous system environment and participate in the regulation of energy balance and reproduction. To assess the interaction of GnRH neurons and hypothalamic astrocytes during the puberty transition in HFD‐treated mice, female and male mice were divided into three groups according to the diet offered at weaning: 42% high‐fat diet (HFD42%), 60% high‐fat diet (HFD60%), or regular diet (CHOW). The effects of HFD on reproductive tissue and fat content during the prepubertal and pubertal transition were assessed. The impact of HFD on astrocyte interaction with GnRH neurons in the medial preoptic area (MPOA) and arcuate/median eminence (ARC/ME) was assessed. HFD anticipated the first signs of puberty in both male and female mice. Furthermore, there was an increase in adipose and reproductive tissue content in early pubertal animals. Remarkably, the anticipation of puberty onset in females treated with HFD was associated with an increase in the astrocyte apposition on GnRH neurons in the MPOA. Also, there was an increase in astrocyte apposition on GnRH neurons and their fiber projections in the ARC/ME. This study suggests that the HFD‐induced anticipation of puberty seems to be, at least partially, mediated by an increase in the morphological association between astrocytes and GnRH neurons in both the MPOA and ARC/EM, which may increase the excitability of GnRH neurons.

## Graphical abstract

This study suggests that the HFD‐induced anticipation of puberty seems to be, at least partially, mediated by an increase in the interaction between astrocytes and GnRH neurons in both the MPOA and ARC/EM, which may increase the excitability of GnRH neurons.

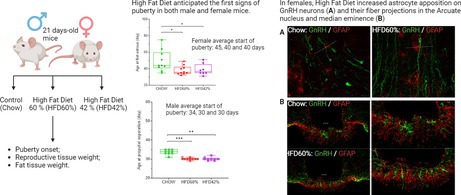

## 1 INTRODUCTION

Puberty is the transition toward the reproductive maturation between childhood and adolescence/adult life, which involves complex biological processes that culminate in the development of secondary sexual characteristics, growth spurt, and reproductive capacity, as well as behavioral changes.^1^ Several studies have revealed that not only neuroendocrine factors, but also complex interactions between genetic components and epigenetic, environmental, and especially nutritional cues, are involved in determining the onset of puberty.^2^, ^3^, ^4^, ^5^ However, the understanding of the events that govern this physiological process still needs to be elucidated.

Puberty is associated with the activation of neurons that secrete gonadotropin‐releasing hormone (GnRH) which, in rodents, are located in the medial preoptic area (MPOA). Secretion of GnRH occurs at the median eminence (ME) and it is transported to the adenohypophysis, promoting the stimulation of follicle‐stimulating hormone (FSH), and luteinizing hormone (LH) secretion to the systemic circulation. Secreted gonadotropins act directly on the gonads, enabling the biosynthesis of sexual steroids, and reproductive maturation.^6^, ^7^ Among the factors that interfere in the onset of puberty, obesity has been one of the most prominent in recent decades. Several epidemiological studies have demonstrated the direct relationship between the increase in childhood obesity rates and the anticipation of puberty,^8^, ^9^, ^10^, ^11^, ^12^ showing that overweight may trigger the permissive neuroendocrine events at the beginning of sexual maturation.^13^ The relationship between obesity and anticipation of puberty is also seen in rodents fed a high‐fat diet (HFD). In these animals, the anticipation of the vaginal opening, the first estrus, and the beginning of reproductive cyclicity in relation to controls was observed.^14^, ^15^, ^16^, ^17^

In recent years, several studies have pointed out the interaction of non‐neuronal cell types, especially the astrocytes, in controlling the hypothalamic neuroendocrine circuits.^18^, ^19^ However, the relationship between the effects of obesity and the astrocytic modulation on the neuroendocrine control of puberty onset^20^ has not been fully established. Hypothalamic inflammation has been reported in diet‐induced obesity.^21^, ^22^, ^23^ Astrocyte hypertrophy and reactive astrogliosis, with an increase in specific structural proteins, such as glial fibrillary acidic protein (GFAP) and vimentin^24^, ^25^ can be induced by HFD. Furthermore, in vitro studies using primary cell culture of astrocytes have demonstrated that saturated fatty acids increase the activity of TLR4 (or Toll‐4 type receptor) and inflammatory response and change the sensitivity to leptin and insulin.^23^, ^26^, ^27^, ^28^

Interestingly, the saturated fatty acids serve as TLR4 ligands that trigger the cascade of production of pro‐inflammatory cytokines such as tumor necrosis factor‐α (TNFα) and interleukin‐6 (IL‐6),^29^, ^30^ and this production, in response to astrocytic activation, also favors the production of prostaglandin E2 (PGE2), an important gliotransmitter that is a fundamental participant in the modulation of GnRH neurons and reproductive function.^31^, ^32^, ^33^, ^34^, ^35^, ^36^, ^37^ Therefore, the astrocytes have a great contribution to the maintenance of the excitability of GnRH neurons.^38^, ^39^

Considering that (1) childhood obesity is related to the anticipation of puberty, which is also observed in HFD administration animal models; (2) the astrocytes are dynamic cells that respond to changes in the CNS environment, such as obesity; and (3) the astrocytes have a great contribution to the maintenance of the excitability of GnRH neurons, the hypothesis of the present work is that the high‐fat diet (HFD) triggers changes in the activity and morphology of astrocytes that are linked to the anticipation of puberty. In the present work, we sought to evaluate the effects of high‐fat diet in male and female mice and astrocytic density and apposition on GnRH neurons in the early puberty model.

## 2 MATERIALS AND METHODS

### 2.1 Animals

Male and female C57BL/6J mice were housed in ventilated shelves acclimated to a controlled ambient temperature of 23 ± 2°C, under a 12 h light/dark cycle (lights on 6:00–18:00 h) with tap water and diet ad libitum. All experimental procedures were approved by the Ethics Committee on the Use of Animals of Ribeirão Preto Medical School, University of Sao Paulo (205/2018) and adhere to the current NIH Guidelines (8th edition, 2011).

### 2.2 Diet

After weaning, the animals received a high‐fat diet with 60% fat (HFD60%; fat: 60% kcal; protein: 20% kcal; carbohydrate: 20% kcal; energy density: 5.21 kcal/g; D12492, Research Diets Inc.), a high‐fat diet with 42% fat (HFD42%; fat: 42% kcal; protein: 15.2% kcal; carbohydrate: 42.7% kcal; energy density: 5 kcal/g; TD.88137, Teklad Global Diet), or a regular diet (CHOW; fat: 10% kcal; protein: 20% kcal; carbohydrate: 70% kcal; energy density: 3.85 kcal/g; Nuvital).

### 2.3 Experimental protocols

#### 2.3.1 Effects of high‐fat diet on pubertal maturation and body weight

After weaning (21 days after birth), female and male mice were divided into three groups: HFD42%, HFD60%, or CHOW. These groups were daily monitored to determine the first pubertal maturation indices. In females, we evaluated the vaginal opening (indicating the onset of puberty) and, thereafter, daily vaginal smears were assessed to analyze the first estrus (indicating the first ovulation).^40^ In males, the day of complete preputial separation was the parameter used to identify the onset of puberty.^41^

On the experimental day, animals were previously exposed to 1% isoflurane before euthanasia by decapitation to perform the collection of the reproductive tissues (uterus and ovary in females and seminal vesicle and testis in males) and adipose tissues (retroperitoneal and perigonadal).

#### 2.3.2 Effect of high‐fat diet on astrocytic appositions in GnRH neurons in the MPOA and ARC/ME in females during the pubertal transition

A subset of HFD60% and CHOW treated females was euthanized in the pubertal transition period (PND32) for the brain collection. The hypothalamic sections were obtained for the immunofluorescence protocol for GnRH and GFAP labeling to assess the astrocyte morphology and its appositions on GnRH neurons in the MPOA and ARC/ME.

### 2.4 Perfusion

The animals were anesthetized with a solution of ketamine (80–100 mg/kg) and xylazine (6–10 mg/kg); perfusion was performed through the infusion, via the thoracic aorta, of 20 mL of sterile isotonic saline (0.15 M NaCl) and heparinized solution (dilution 1:80), followed by the infusion of 40 mL of 4% formalin (Sigma). After the perfusion, the brains were removed, post‐fixed in 4% formalin for 4 h at 4°C, and cryoprotected in 30% sucrose diluted in 0.01 M PBS at 4°C for 4 days. After cryoprotection, 30 μm coronal sections were obtained in a cryostat (Leica Model CM1850) from the entire rostrum‐caudal extension of the medial preoptic area (MPOA, Bregma 0.74 to −0.58 mm) and arcuate nucleus and median eminence (ARC/ME, Bregma −1.22 to −2.70 mm), according to the coordinates of the atlas of Franklin and Paxinos.^42^ The sections were stored in a cryoprotectant solution at −20°C until the immunofluorescence procedure.

### 2.5 Immunofluorescence and microscopy imaging

*Double labeling for GnRH and GFAP*: The coronal sections were rinsed in 1X Tris buffer to remove the cryoprotectant solution. Blocking of nonspecific binding, as well as primary antibody incubations, were performed in solution using 10% normal horse serum (Jackson Immuno Research Laboratories Inc., JACK‐008‐000‐121) and Triton X diluted in 1X Tris buffer. The antibody anti‐GnRH (Rabbit Anti‐GnRH, from T. Nett laboratory) was used at a concentration of 1:10,000, while the primary antibody for the identification of astrocytes (Mouse Anti‐GFAP, Sigma g3893) was used in a concentration of 1:400. Incubation in primary antibodies was carried out at 4°C for 48 h. On the second day, sections were incubated for 1 h with a biotinylated secondary antibody (Donkey Anti‐Rabbit) at a concentration of 1:200. After this procedure, the sections were rinsed and then incubated with secondary antibody (Alexa 647—Donkey Anti‐Mouse, Abcam CD715.605.151) at a concentration of 1:250. Amplification of GnRH signal by Alexa 488 conjugated with Streptavidin [1:250], diluted in 10% blocking solution, was obtained by further incubation for 1 h. At the end of the procedures, the sections were rinsed in 1X Tris buffer, mounted onto gelatin‐subbed slides, and coverslipped with Fluoromont®.

The immunofluorescence images were obtained using an optical microscope (DM4500 B, Leica), equipped with 20× and 40× objectives, coupled with an image analysis system (LAS V3.8, Leica) and a multiphoton laser scanning microscope (LSM 780—Axio Observer—Zeiss) equipped with a 63× objective. The sections containing the MPOA and ARC/ME were analyzed using ImageJ software.

To identify the appositions of astrocytes on GnRH neurons in the MPOA, an area of 100 μm was selected close to the GnRH‐soma. The data obtained by ImageJ measurement profile was defined in an area of 20 μm from the selection (based on the presence of GnRH neuron), as shown in Figure 4B,C. This method to analyze the interactions of astrocytes and GnRH neurons was based on the analysis of points of connectivity between these two cells in the MPOA area previously reported in the literature.^43^

### 2.6 Statistical analysis

The effect of 42% and 60% HFD on puberty onset and body weight was determined by one‐way ANOVA followed by Dunnett's post‐test. The effects of diet (CHOW or HFD60%) and age (during prepubertal and pubertal transition) on reproductive tissue and fat content in female and male mice were determined by two‐way ANOVA followed by Tukey post‐hoc test. To assess the GnRH and astrocyte (GFAP) interaction, the total fluorescence intensity within the defined area was quantified, and the area under the curve (AUC) was calculated as a semi‐quantitative representation of the functional interaction between GnRH and astrocytes. For each animal (*n* = 3), three neurons or connections were analyzed, and the obtained AUC values were used for statistical comparisons. The Mann–Whitney test was applied to compare the intensities between groups, with a significance level of *p* < .05. This test was used to evaluate the statistical differences between the groups in the fluorescence intensity values of GnRH and GFAP in the control (CHOW) and HFD groups. To assess the qualitative interaction between the fluorescent signals of GnRH and astrocytes labeled with GFAP, regions of interest (ROIs) of 20 μm were defined along the neuronal projections, based on the midpoint of GnRH projections, where overlapping fluorescent signals of both proteins were observed. All statistical analyses and graphs were performed using GraphPad Prism software 8.0 GraphPad Software, San Diego California USA.

## 3 RESULTS

### 3.1 HFD advances puberty onset in female and male mice

**FIGURE 1:**
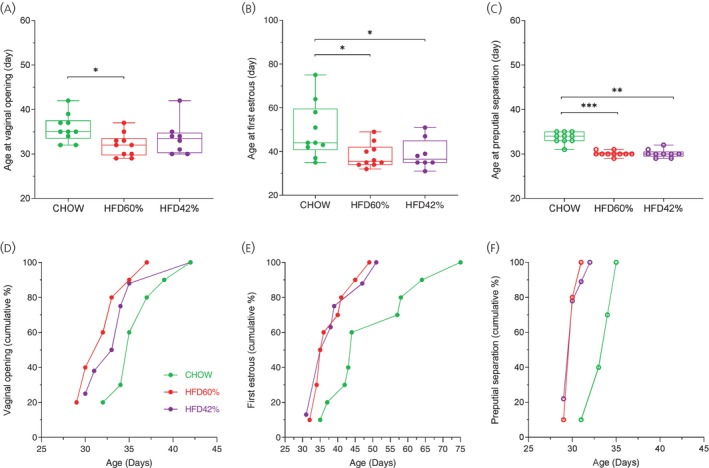
High fat diet (HFD) advances puberty onset in female and male mice. Mice were fed from weaning with 42% high‐fat diet (HFD42%), 60% high‐fat diet (HFD60%), or regular diet (CHOW). (A) Age at vaginal opening (VO, *n* = 8–10). (B) Age at first estrous (*n* = 8–10). (C) Age at preputial separation (*n* = 9–10). Cumulative percentage of females showing VO (D) and first estrous (E). Cumulative percentage of males showing preputial separation (F). Data are shown as mean ± SEM and were analyzed by one‐way ANOVA followed by Dunnett's post‐hoc test. Significance was defined as *p* < .05 (*) in relation to the control group (CHOW).

Figure 1 shows the effect of postweaning feeding with HFD (HFD60% or HFD42%) on puberty onset in female (A and B) and male mice (C). In female mice, a significant anticipation of age at vaginal opening was observed in animals fed with HFD (*F*_[2, 25]_ = 3.582; *p* = .0429), although this anticipation was evidenced only in the HFD60% group when compared to CHOW animals (Figure 1A). Sixty percent of females fed with HFD60% exhibited vaginal opening by PND32 (Figure 1D), whereas 60% of the CHOW group reached vaginal opening by PND35. On the other hand, both HFD60% and HFD42% females anticipated the age at first estrous when compared to the CHOW group (*F*_[2, 25]_ = 4.627; *p* = .0195; Figure 1B). When comparing the progression of the first estrous, we observed that 50% of both HFD‐fed and CHOW‐fed females experienced their first estrous by PND35 and PND43, respectively. In male mice, both HFD‐fed groups anticipated the age at preputial separation (*F*_[2, 26]_ = 46.07; *p* < .0001), in relation to the CHOW group (Figure 1C). This represents 80% of HFD‐fed males showing preputial separation by PND30, in contrast to 80% of the CHOW group by PND34. To assess whether changes in reproductive organs and fat tissues precede the onset of puberty, that is, VO in females and preputial separation in males, we evaluated several parameters 5 days before these first signals. In females, as the mean age of VO in the HFD60% group was PND32, the analyses were carried out on PND27 and compared to the CHOW group at the same age. The same assessments were carried out on PND32, the day of puberty onset in the HFD60% group.

### 3.2 Effects of HFD on body weight at puberty onset in female and male mice

**FIGURE 2:**
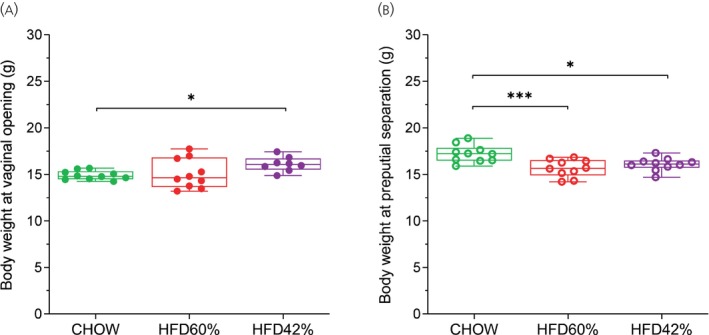
Effects of HFD on body weight at puberty onset in female (A, *n* = 8 to 10) and male (B, *n* = 10) mice. Data are shown as mean ± SEM and were analyzed by one‐way ANOVA followed by Dunnett's post‐hoc test. Significance was defined as *p* < .05 (*) in relation to the control group.

Figure 2 shows the body weight of female (A) and male (B) mice on the day of VO and preputial separation, respectively, to determine the relationship between body weight and puberty onset. In female mice (Figure 2A), despite both HFD groups having precociously presented puberty signals (see Figure 1A,B), the ANOVA one‐way analysis did not detect the difference between CHOW and HFD groups on body weight on the day of VO (*F*_[2, 25]_ = 3.134; *p* = .0610). In males, the body weight of HFD groups was lower than the control group (CHOW) on the day of preputial separation (*F*_[2, 27]_ = 9.073; *p* = .0010; Figure 2B), even though these animals had anticipated the onset of puberty. The mean body weight gain (Figure S1A,B) and the body weight gain over 13 weeks of age were also assessed (Figure S2A,B).

### 3.3 Effects of HFD on reproductive tissue and body fat content during prepubertal and pubertal transition in female and male mice

**FIGURE 3:**
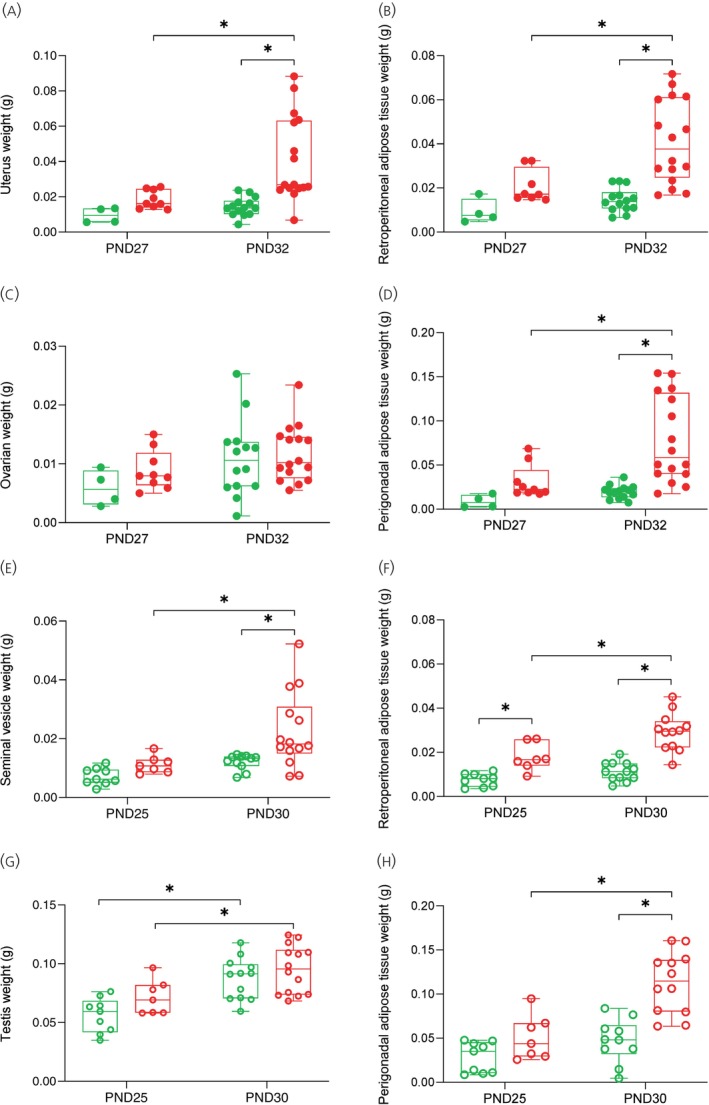
Effects of HFD on reproductive tissue and body fat weight during prepubertal and pubertal transition in female and male mice. Data of prepubertal female mice (PND27, *n* = 4–9) or in the pubertal transition (PND32, *n* = 14–16) are represented in the figures A, B and C, D. Data of prepubertal male mice (PND25, *n* = 7–9) and in the pubertal transition (PND30, *n* = 12–14) are represented in the figures E, F and G, H. Data from CHOW are shown in green, and data from HFD 60% are shown in red. Data are presented as mean ± SEM. Significance was defined as *p* < .05 (*). Statistical analysis was performed using two‐way ANOVA followed by Tukey's post‐hoc test.

To assess whether changes in reproductive organs and fat tissues precede the onset of puberty, that is, VO in females and preputial separation in males, we evaluated several parameters 5 days before these first signals. In females, as the mean age of VO in the HFD60% group was PND 32, the analyses were carried out on PND 27 and compared to the CHOW group at the same age. The same assessments were carried out on PND 32, the day of puberty onset in the HFD60% group. In male mice, as the mean age at preputial separation was PND 30 in the HFD60% group, the analyses were carried out on PND 25 as well as on the day of the onset of puberty, PND30. The effects of diet and age on tissue content were measured, and these data are shown in Figure 3.

HFD induced an increase in the weight of both reproductive organs and adipose tissue during the pubertal transition in both male and female mice. There was a significant effect of age on the uterus weight (Figure 3A; *F*_[1, 39]_ = 6.331, *p* = .0161) and diet (*F*_[1, 39]_ = 10.64, *p* = .0023), with no interaction between both parameters. The uterine weight of females fed with HDF60% on PND32 was greater than that of the CHOW group (HFD60%: PND32 vs. Chow:PND32; *p* = .002). Ovarian weight (Figure 3C) was modified by age (*F*_[1, 39]_ = 4.834, *p* = .0339), but not by diet, with an interaction between both parameters.

In males, the two‐way ANOVA test indicated that age (Figure 3E; *F*_[1, 37]_ = 10.09, *p* = .0030) and diet (*F*_[1, 37]_ = 8.021, *p* = .0074) affected the seminal vesicle weight. The post‐hoc analysis revealed that seminal vesicle weight was greater in the HFD60% group when compared to all other groups only at PND30, age of puberty onset in this group (CHOW:PND30 vs. HFD60%:PND30, *p* = .0119 and HFD60%:PND30 vs. HFD60%:PND25, *p* = .0191). The increase in testis weight was also influenced by age (Figure 3G; *F*_[1, 38]_ = 23.72, *p* < .0001) and diet (*F*_[1, 38]_ = 4.301, *p* = .0449). No interaction between the two parameters was observed across any of the weights analyzed.

In relation to the retroperitoneal and perigonadal adipose weight of female mice (Figure 3B,D), there was a significant effect of age (retroperitoneal: *F*_[1, 38]_ = 7.750, *p* = .0083; perigonadal: *F*_[1, 39]_ = 6.825, *p* = .0127) and diet (retroperitoneal: *F*_[1, 38]_ = 17.33, *p* = .0002; perigonadal: *F*_[1, 39]_ = 13.24, *p* = .0008). The weight of both adipose tissues was greater in females fed with HFD60% only at the onset of puberty (day 32) when compared to all other groups, with no significant interaction between the two factors. The effect of diet on retroperitoneal tissue was: CHOW:PND32 versus HFD60%:PND32, *p* < .0001; in perigonadal tissue the effect was: CHOW:PND32 versus HFD60%:PND32, *p* < .0001. The effect of HFD was also affected by diet exposure. The weight of retroperitoneal tissue was greater at PND32 (HFD60%:PND27 vs. HFD60%:PND32, *p* = .0043) as well as on the perigonadal tissue (HFD60%:PND27 vs. HFD60%:PND32, *p* = .0049).

As in females, there was an effect of HFD duration (Figure 3F; *F*_[1, 36]_ = 15.37, *p* = .0004; Figure 3H; *F*_[1, 34]_ = 20.21, *p* < .0001) and diet (Figure 3G; *F*_[1, 36]_ = 52.80, *p* < .0001; Figure 3H; *F*_[1, 34]_ = 24.15, *p* < .0001) on the weight of adipose tissue retroperitoneal (Figure 3F) and perigonadal (Figure 3H) in males. Additionally, a significant interaction between the factors was observed for the perigonadal adipose tissue (Figure 3H; interaction: *F*_[1, 34]_ = 5.945, *p* = .0201) while no interaction was detected for the retroperitoneal adipose tissue (Figure 3F).

The post‐hoc analysis indicated that HFD60% induced a significant increase in both adipose tissue weights at PND30 when compared to their control groups (Figure 3F: CHOW:PND30 vs. HFD60%:PND30, *p* < .0001 and Figure 3H: CHOW:PND30 vs. HFD60%:PND30, *p* < .0001). This effect of HFD was observed earlier at PND25 only in retroperitoneal adipose tissue (Figure 3F: CHOW:PND25 vs. HFD60%:PND25, *p* = .0085). The effect of HFD was also influenced by diet exposure as adipose tissue weight was greater at PND30 than at PND25 (Figure 3F: HFD60%:PND25 vs. HFD60%:PND30, *p* = .0016 and Figure 3H: HFD60%:PND25 vs. HFD60%:PND30, *p* = .0002).

### 3.4 Effect of HFD on astrocyte appositions on GnRH neurons in the MPOA and ARC/ME in females during the pubertal transition

**FIGURE 4:**
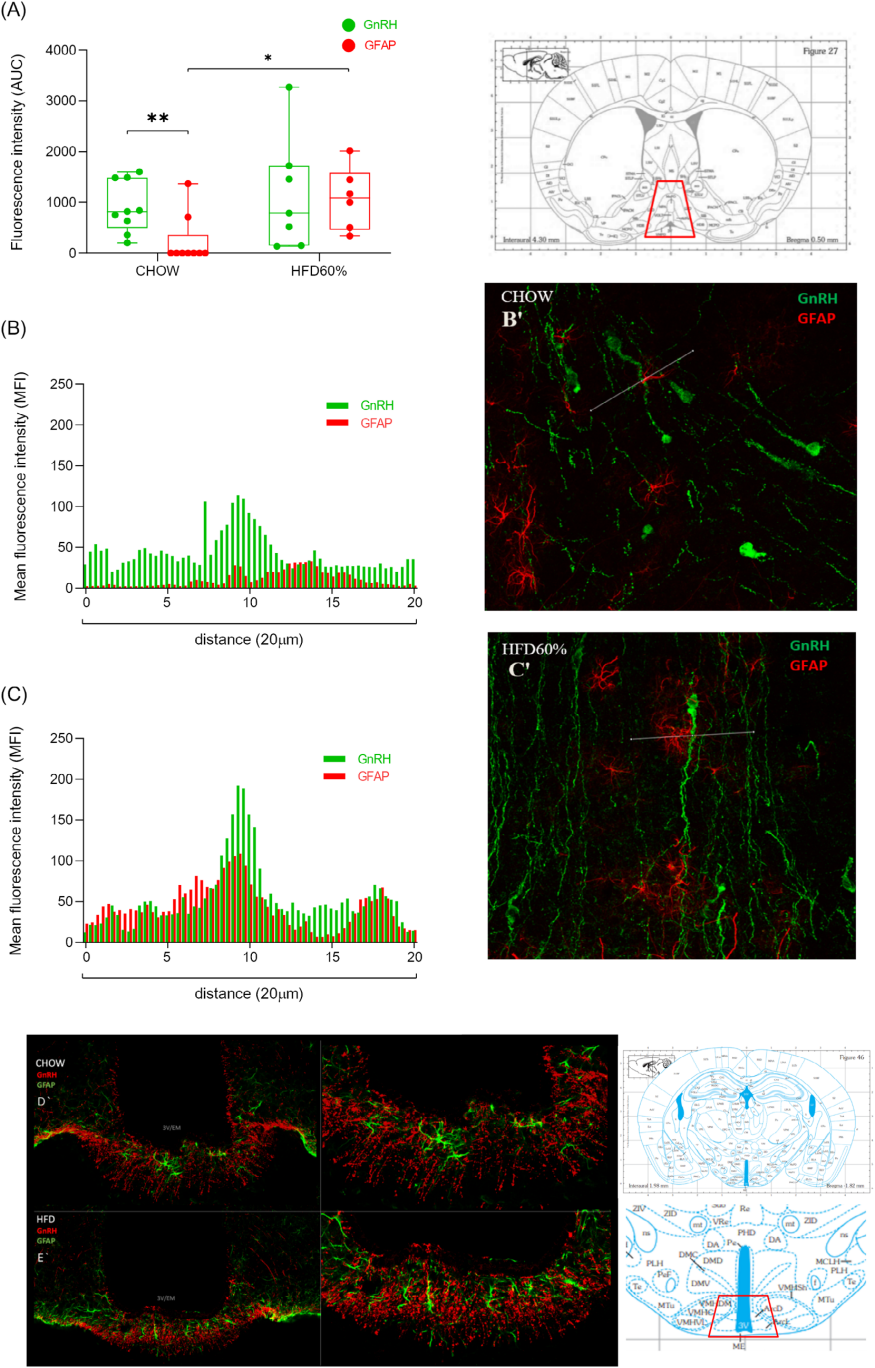
Effect of high‐fat diet on astrocytic appositions in GnRH neurons in the MPOA and ARC/ME in females during the pubertal transition. (A) Fluorescence intensity (AUC, *n* = 7–9. MPOA) of GnRH neurons and astrocytes (GFAP) analyzed by Mann–Whitney test (diet effect is shown as *). Data are shown as mean ± SEM. Schematic graphic (B: CHOW and C: HFD60%) between GnRH and GFAP fluorescent peaks measured in 20 μm long (ROI) considering the middle point of GnRH presence. Representative photomicrography of MPOA (B′: CHOW and C′: HFD60%) and ARC/ME (D: CHOW and E: HFD60%. 3 V: 3rd), showing GnRH and GFAP labeling. The images are the maximum intensity projection of an individual optical plane. Representative mouse brain plates were obtained from Paxinos and Franklin.^42^

The semiquantitative analysis of the effect of HFD60% on astrocytes and GnRH neurons (fluorescence intensity measure) in the MPOA is illustrated in Figure 4A–C. The results from the Mann–Whitney tests (Figure 4A) showed no significant difference in fluorescence intensity between GnRH neurons in CHOW and HFD60% groups (*p* = .9182). However, a significant increase in GFAP fluorescence intensity was observed in the HFD60% group compared to the CHOW group (*p* = .0224), indicating a diet‐induced effect on astrocyte activation. In the analysis of fluorescence intensity between GnRH and GFAP cells within each group, a significant difference was observed in the CHOW group (*p* = .0032), with GnRH showing higher fluorescence intensity compared to GFAP. Interestingly, no significant difference was found between GnRH and GFAP fluorescence intensity in the HFD60% group (*p* = .8048). This lack of difference in the HFD group suggests an increase in GFAP immunoreactivity in early‐puberty animals, potentially reflecting enhanced astrocyte activity under this dietary condition.

To illustrate possible appositions, a graphic composed of a mean fluorescence intensity (MFI) between GnRH and GFAP was performed in samples from CHOW and HFD60% groups (Figure 4B,C, respectively, in a distance measured between interactions of 20 μm, considering the middle point of GnRH labeling). The fluorescence images from the MPOA area (Figure 4, B′ and C′) show a model of GnRH and GFAP analysis in CHOW and HFD60% obtained in female mice at PND32. Figure 4D,E illustrate the presence of both cells in the ARC/ME nucleus, showing a clear increase in the GFAP appositions on GnRH terminals in HFD60%‐fed females at PND32.

## 4 DISCUSSION

The present study reinforces the idea that the high‐fat diet anticipates the first signs of puberty in male and female mice. Remarkably, the anticipation of puberty onset was associated with an increase in the apposition of astrocytes on GnRH neurons in the MPOA, as well as an increase in GFAP immunoreactivity in the median eminence in females. Furthermore, our results suggest that there is a sex‐specific difference regarding the effects of HFD on body weight at the beginning of puberty, since the mean weight gain was not different from the CHOW group in females; the males presented a slightly lower weight in relation to their controls at puberty onset.

It is well known that childhood obesity has been one of the factors mostly related to the advance of pubertal maturation.^8^, ^9^, ^10^, ^11^, ^12^ In our study, ingestion of 42% or 60% HFD from weaning was able to anticipate the signs of puberty in males and females, as well as the presence of the first estrus. The link between HFD and obesity and the anticipation of puberty onset was observed in other studies in rodents.^14^, ^15^, ^16^, ^17^ We found in females that there was an association in body weight gain and puberty onset; however, in males, this association was not evident, since the animals fed with HFD had lower body weight compared to controls on the day of preputial separation. Sexually dimorphic mechanisms, such as leptin and kisspeptin, may contribute to this difference, as leptin is associated with puberty in girls^44^, ^45^ but not clearly in boys,^46^ and kisspeptin expression is higher in females, potentially leading to earlier puberty onset.^47^ Thus, hormonal and metabolic differences may influence how body weight affects puberty, and in males, metabolic changes induced by HFD may be sufficient to advance puberty regardless of weight gain, though this remains underexplored.

Pioneering studies conducted in humans^48^ and rodents^49^ established the link between reproductive maturity and a critical body weight. But, more recently, the anticipation of puberty after HFD was shown to occur regardless of changes in body weight in female mice,^50^ while the association between body weight and early reproductive function is still uncertain in boys^51^, ^52^ and male mice.^53^, ^54^, ^55^

It is well known that white adipose tissue plays an important role in the maintenance and modulation of metabolism, and, in females, obesity induced by a high‐fat diet seems to be able to interfere in adult ovary function, as folliculogenesis.^56^ This response occurs through the paracrine action from perigonadal and abdominal (retroperitoneal) adipose tissue in the ovary, since the adult perigonadal removal exerts a negative effect on ovarian steroidogenesis, as well as lipid, adipokines, and growth factors that are important to follicular development in adults.^57^, ^58^ In our study, the increases in adipose and reproductive tissue content observed in animals fed with HFD60% may mirror the early onset of puberty.

In addition to the peripheral tissues, it has been shown that obesity is also associated with inflammation in the hypothalamus.^21^, ^22^, ^23^ These changes lead to a state of metabolic stress that promotes the synthesis and release of pro‐inflammatory mediators, which lead to the suppression of central leptin signaling, resulting in resistance to this hormone, hyperleptinemia, and consequent dysregulation of food ingestion and energy balance.^28^, ^59^, ^60^ However, it should be pointed out that, in contrast to prolonged and sustained exposure to an obesogenic diet, leptin action in the hypothalamus is maintained and this hormone can mediate the increase of kisspeptin expression after short‐term exposure to HFD and advance in the puberty onset.^17^

Hypothalamic astrocytes express leptin receptors^60^, ^61^ and leptin acts on astrocytes, contributing to the regulation of energy metabolism and undergoing morphological changes when in a constant state of positive energy balance, as in obesity. Among these changes observed in obesity models are astrocytic hypertrophy and reactive astrogliosis.^24^, ^25^, ^62^, ^63^

We found a clear augmentation of interaction of astrocyte processes in GnRH neurons in females fed with HFD60% during pubertal transition. It is known that astrocytes are required for reproductive function,^34^, ^43^, ^64^ and part of these actions come from the synthesis of PGE2 in the hypothalamic astrocytes,^64^, ^65^ which is likely to be the link between the astrocyte modulation in the GnRH neuron activity.^64^, ^66^, ^67^, ^68^, ^69^ The PGE2 secreted by astrocytes acts directly on GnRH neurons through interaction with its receptor (PTGER2) located in the neuronal membrane, which triggers intracellular mechanisms that stimulate the opening of non‐selective cation channels, inducing membrane depolarization and increasing the rate of firing of these neurons.^70^ The importance of PGE2 for reproduction is also recognized in early puberty. Rats with hypothalamic deficiency of PGE2 synthesis present delayed puberty onset.^35^ As puberty approaches, there is an increase in estradiol concentrations produced by ovarian maturation, which favors the synthesis and secretion of hypothalamic PGE2. Together, these events can induce preovulatory secretion of the first peak of GnRH and LH during puberty onset.^34^

Interestingly, the levels of circulating ovarian steroids also modify the astrocytic coverage and the influence of these cells on GnRH neurons in the control of reproduction.^71^ Witkin et al.^72^ demonstrated in ovariectomized monkeys that the treatment with estradiol increases the interaction of astrocytes with GnRH neurons, which may be related to the stimulatory role of estradiol in GnRH secretion during the estrous cycle. In addition to these mechanisms, recent findings indicate that kisspeptin also influences astrocytes, which, in turn, could impact GnRH neurons and the reproductive responses to metabolic stress, such as those induced by HFD.^73^ The changes in astrocyte morphology can be modified by a synaptic adhesion molecule (SynCAM) that acts by regulating the alignment of synaptic junctions and is expressed both in neurons and in hypothalamic astrocytes. Its entire functioning provides the activity of the central reproductive system, as well as the onset of puberty, since female mice that exhibit alterations in SynCAM actions have a delay in puberty onset, in addition to disturbances in the estrous cycle and fertility.^43^, ^74^ Further investigation will be necessary to establish the molecular mediators from astrocytes in the activation of GnRH neurons during puberty transition, as well as to provide direct evidence of enhanced interaction between these cells. While immunofluorescence allowed us to assess astrocyte apposition on GnRH neurons, a quantitative analysis would provide additional insights and should be explored in future studies. To confirm this hypothesis, future studies employing electron microscopy to better assess astrocyte apposition to GnRH neurons, as well as electrophysiological or optogenetic approaches to identify the underlying mechanisms, will be required.

Together, our findings show that: (1) the early puberty onset induced by HFD diet is associated with a greater amount of retroperitoneal as well as perigonadal adipose tissue rather than body weight and (2) HFD‐induced early puberty onset is associated with an increased astrocytic apposition on MPOA‐GnRH neurons and increased GFAP immunoreactivity observed in the ARC/ME region. This data reinforce the role of astrocytes on the early puberty onset induced by HFD.

## AUTHOR CONTRIBUTIONS

**Isabelle Rodrigues‐Santos:** Conceptualization; methodology; writing – original draft; writing – review and editing; investigation. **Raoni Conceição Dos‐Santos:** Methodology; investigation. **Aline de Jesus:** Methodology; investigation. **Rafael Appel Flores:** Methodology; investigation. **Roberta Ribeiro Costa Rosales:** Methodology; investigation. **Izabela Facco Caliman:** Methodology; investigation. **Janete A. Anselmo‐Franci:** Methodology; investigation. **José Antunes‐Rodrigues:** Methodology; supervision; project administration; resources; investigation; funding acquisition. **Lucila Leico K. Elias:** Conceptualization; methodology; supervision; project administration; resources; writing – original draft; writing – review and editing; investigation; funding acquisition.

## FUNDING INFORMATION

This study was financially supported by a grant from the São Paulo Research Foundation 2018/18071‐5 and the Brazilian National Council for Scientific and Technological Development 304791/2018‐0 awarded to Lucila Leico K. Elias and CNPq169914/2018‐6 awarded to Isabelle Rodrigues‐Santos and FAEPA (Fundação de Apoio ao Ensino, Pesquisa e Assistência).

## CONFLICT OF INTEREST STATEMENT

The authors declare no conflicts of interest.

## PEER REVIEW

The peer review history for this article is available at https://www.webofscience.com/api/gateway/wos/peer-review/10.1111/jne.70029.

## ETHICS STATEMENT

All procedures were approved by the Committee for Animal Care and Use (CEUA‐FMRP number 205/2018) of the Ribeirão Preto Medical School, University of São Paulo.

## Supporting information


**Figure S1.** Average food intake between groups. The animals were distributed in two animals per cage, and food intake was measured weekly. Data are shown as mean ± SEM and were analyzed by one‐way ANOVA followed by Dunnett's post‐hoc test. Significance was defined as *p* < .05 (*) in relation to the control group.
**Figure S2.** Body weight gain over 13 weeks of life. Mice were fed a regular diet (CHOW), a 60% high‐fat diet (HFD60%) or 42% high‐fat diet (HFD42%). (A) Weight gain in females (*n* = 6–10) and (B) weight gain in males (*n* = 10). Data are shown as mean ± SEM and were analyzed by Mixed‐effects followed by Dunnett's test. Significance was defined as *p* < .05 (*) to CHOW versus HFD42% and (#) to CHOW versus HFD60%.

## Data Availability

The data that support the findings of this study are available from the corresponding author upon reasonable request.
