# Supplementary figures and images for: Increase of astrocyte apposition on GnRH neurons in early puberty onset induced by high fat diet
Source: J Neuroendocrinol. 2025 Apr 15;37(8):e70029. doi: 10.1111/jne.70029 (PMC12358207; doi:10.1111/jne.70029)

Supplementary Figure 1

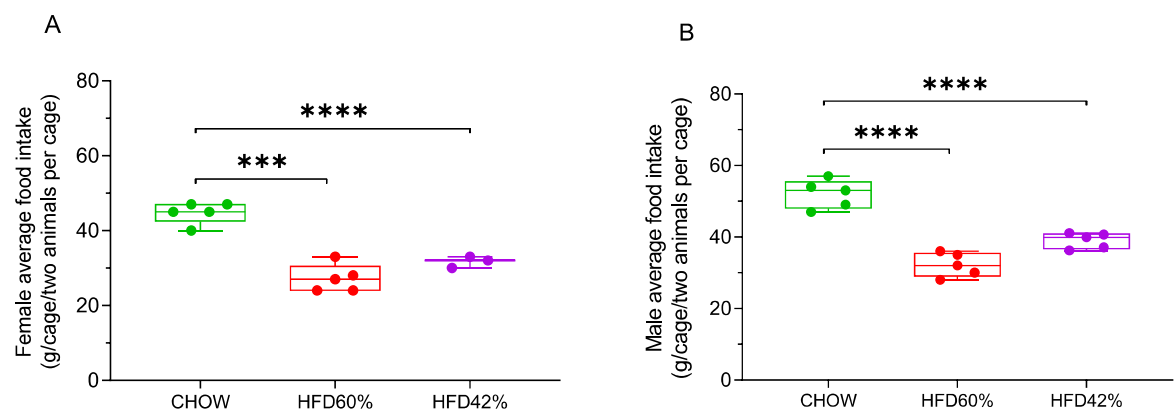

Supplementary figure 2:

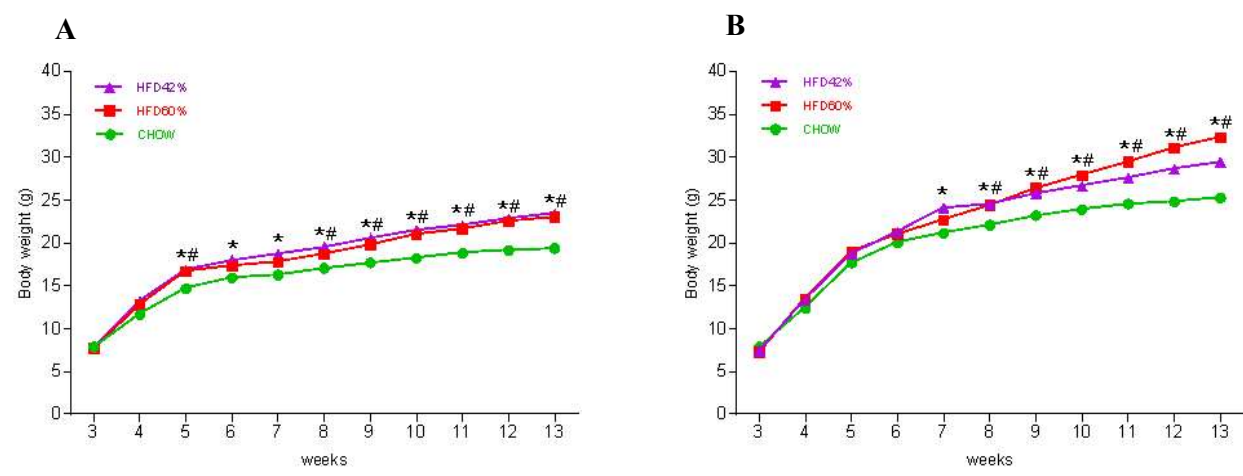

Supplement: Supplementary file 1 — Figure S1. Average food intake between groups. The animals were distributed in two animals per cage, and food intake was measured weekly. Data are shown as mean ± SEM and were analyzed by one‐way ANOVA followed by Dunnett's post‐hoc test. Significance was defined as p < .05 (*) in relation to the control group. Figure S2. Body weight gain over 13 weeks of life. Mice were fed a regular diet (CHOW), a 60% high‐fat diet (HFD60%) or 42% high‐fat diet (HFD42%). (A) Weight gain in females (n = 6–10) and (B) weight gain in males (n = 10). Data are shown as mean ± SEM and were analyzed by Mixed‐effects followed by Dunnett's test. Significance was defined as p < .05 (*) to CHOW versus HFD42% and (#) to CHOW versus HFD60%. [file JNE-37-e70029-s001.pdf]
